# Initial Japanese Multicenter Experience and Age-Related Outcomes Following Left Atrial Appendage Closure: The OCEAN-LAAC Registry

**DOI:** 10.1016/j.jacasi.2022.11.003

**Published:** 2023-02-14

**Authors:** Masahiko Asami, Toru Naganuma, Yohei Ohno, Tomoyuki Tani, Hideharu Okamatsu, Kazuki Mizutani, Yusuke Watanabe, Masaki Izumo, Mike Saji, Shingo Mizuno, Hiroshi Ueno, Shunsuke Kubo, Shinichi Shirai, Masaki Nakashima, Masanori Yamamoto, Kentaro Hayashida, OCEAN-LAAC Investigators

**Affiliations:** a Division of Cardiology, Mitsui Memorial Hospital, Tokyo, Japan; b Department of Cardiology, New Tokyo Hospital, Chiba, Japan; c Department of Cardiology, Tokai University School of Medicine, Kanagawa, Japan; d Department of Cardiology, Sapporo East Tokushukai Hospital, Hokkaido, Japan; e Department of Cardiology, Saiseikai Kumamoto Hospital, Kumamoto, Japan; f Department of Cardiology, Kinki University School of Medicine, Osaka, Japan; g Department of Cardiology, Teikyo University School of Medicine, Tokyo, Japan; h Department of Cardiology, St Marianna University School of Medicine, Kanagawa, Japan; i Department of Cardiology, Sakakibara Heart Institute, Tokyo, Japan; j Department of Cardiovascular Medicine, Toho University Faculty of Medicine, Tokyo, Japan; k Department of Cardiology, Shonan Kamakura General Hospital, Kanagawa, Japan; l Department of Cardiology, Toyama University Hospital, Toyama, Japan; m Department of Cardiology, Kurashiki Central Hospital, Okayama, Japan; n Department of Cardiology, Kokura Memorial Hospital, Fukuoka, Japan; o Department of Cardiology, Sendai Kousei Hospital, Miyagi, Japan; p Department of Cardiology, Toyohashi Heart Center, Aichi, Japan; q Department of Cardiology, Nagoya Heart Center, Aichi, Japan; r Department of Cardiology, Gifu Heart Center, Gifu, Japan; s Department of Cardiology, Keio University School of Medicine, Tokyo, Japan

**Keywords:** age-related outcomes, left atrial appendage closure, left atrial appendage occlusion, multicenter registry, nonvalvular atrial fibrillation

## Abstract

**Background:**

Limited data are available describing left atrial appendage closure (LAAC) and age-related outcomes in Asians.

**Objectives:**

This study summarizes the initial experience with LAAC in Japan and determines age-related clinical outcomes in patients with nonvalvular atrial fibrillation undergoing percutaneous LAAC.

**Methods:**

In an ongoing, prospective, investigator-initiated, multicenter, observational registry of patients undergoing LAAC in Japan, we analyzed short-term clinical outcomes in patients with nonvalvular atrial fibrillation who underwent LAAC. Patients were classified into younger, middle-aged, and elderly groups (≤70, 70 to 80, and >80 years of age, respectively) to determine age-related outcomes.

**Results:**

Patients (n = 548; mean age, 76.4 ± 8.1 years; male, 70.3%) who underwent LAAC at 19 Japanese centers between September 2019 and June 2021 were enrolled in the study, including 104, 271, and 173 patients in the younger, middle-aged, and elderly groups, respectively. Participants had a high-risk of bleeding and thromboembolism with a mean CHADS_2_ score of 3.1 ± 1.3, a mean CHA_2_DS_2_-VASc score of 4.7 ± 1.5, and a mean HAS-BLED score of 3.2 ± 1.0. Device success rates were 96.5% and anticoagulants discontinuation at the 45-day follow-up was achieved in 89.9%. In-hospital outcomes were not significantly different, but major bleeding events during the 45-day follow-up were significantly higher in the elderly group compared to the other groups (younger vs middle-aged vs elderly, 1.0% vs 3.7% vs 6.9%, respectively; *P* = 0.047) despite the same postoperative drug regimens.

**Conclusions:**

The initial Japanese experience with LAAC demonstrated safety and efficacy; however, perioperative bleeding events were more common in the elderly and postoperative drug regimens must be tailored (OCEAN-LAAC [Optimized Catheter Valvular Intervention–Left Atrial Appendage Closure] registry; UMIN000038498)

Unprecedented aging is coming to developed countries, and the number of patients suffering from atrial fibrillation (AF) continues to increase. Patients with AF have a 5-fold increase in stroke risk compared to patients with sinus rhythm.^1^ AF-related stroke events commonly induce severe disabilities.^2^ Thus, AF management focused on stroke prevention is extremely crucial and attracts a great deal of interest. Vitamin K antagonists (VKAs) dramatically reduce the risk of stroke and mortality.^3^ However, patients who take VKAs are more likely to have severe bleeding events, such as hemorrhagic stroke, gastrointestinal bleeding, or other clinically significant bleeding.^4^, ^5^, ^6^, ^7^ Therefore, treatment has shifted from VKAs to direct oral anticoagulants (DOACs), which are associated with fewer bleeding events than VKAs. Unfortunately, DOACs cannot be used in patients with severe chronic kidney disease and are limited by the lack of therapeutic effect monitoring methods and lack of specific neutralizing agents.

Percutaneous left atrial appendage closure (LAAC) prevents AF-related complications in patients with nonvalvular atrial fibrillation (NVAF), and its use is rapidly expanding as an alternative approach to antithrombotic agents.^8^ However, limited data concerning LAAC using the WATCHMAN 2.5 (LAAC device) and WATCHMAN FLX (new-generation LAAC device; Boston Scientific,) devices are available in Japan. Furthermore, the demographics in patients undergoing LAAC is difference according to age, and it may be affected by postoperative drug regimens. In addition, investigation of age-related clinical outcomes is important in seeking optimal postoperative drug regimens for LAAC according to age. Herein, the initial Japanese LAAC outcomes and age-related clinical outcomes in patients with NVAF are reported.

## Methods

### Study design and patient population

The OCEAN-LAAC (Optimized Catheter Valvular Intervention–Left Atrial Appendage Closure) registry, which is registered with the University Hospital Medical Information Network (UMIN000038498), is an ongoing, prospective, investigator-initiated, multicenter, observational registry of patients with NVAF undergoing percutaneous LAAC using either an LAAC device or a new-generation LAAC device in Japan. Nineteen centers in Japan participate in the registry, which was initiated in September 2019. A listing of investigators and sites can be found in Supplemental Table 1. Patients were candidates for LAAC if they had NVAF requiring anticoagulants and a high bleeding risk that met at least one of the following conditions: 1) HAS-BLED score of three or more; 2) multiple histories of traumas due to a fall; 3) a history of diffuse cerebral amyloid angiopathy; 4) taking both an anticoagulant agent and dual-antiplatelet therapy (triple therapy) long-term; and 5) a history of major bleeding of Bleeding Academic Research Consortium (BARC) type 3 or more. The exclusion criteria for the present analysis were as follows: 1) thrombus in the atrium or appendage; 2) prohibition of either insertion of transesophageal echocardiography (TEE) or a catheter due to an anatomical reason; and 3) contraindications for taking anticoagulants or antiplatelet agents. The registry recorded baseline characteristics focusing on risk scores for frail (clinical frailty scale), bleeding (HAS-BLED score), and thromboembolism (CHADS_2_ and CHA_2_DS_2_-VASc scores). Preprocedural and postprocedural imaging assessments (transthoracic echocardiography [TTE] and TEE]), procedural-related data, postprocedural clinical outcomes, and detailed medication data before and after LAAC were also collected in the registry. Data were prospectively collected with the use of a dedicated web-based database (Canon Medical Systems).

In each center, the local multidisciplinary brain-heart team, consisting of cardiac surgeons, interventional cardiologists, neurologists, brain surgeons, and imaging specialists, determined the eligibility for LAAC and the treatment strategy. LAAC was performed according to standard techniques, as previously described.^9^^,^^10^ The local ethics committee of all participating centers approved the study protocol, and all procedures were conducted in accordance with the Declaration of Helsinki. All patients provided informed consent for the intervention and prospective follow-up before participation in this registry.

### Preprocedural imaging assessments

All patients underwent TTE and/or TEE before LAAC. At least 3 consecutive heartbeats were recorded, and data were averaged for the analysis of echocardiographic variables. According to current guidelines by the American Society of Echocardiography and the European Association of Cardiovascular Imaging, 2-dimensional (2D) and 3-dimensional (3D) echocardiographic assessments were performed.^11^ For baseline TEE measurements, ostium diameter and depth of the left atrial appendage (LAA) were recorded at 0°, 45°, 90°, and 135°.^12^ In addition, LAA morphology was assessed.

### Data collection for mandated clinical follow-up

Clinical follow-up was performed at hospital discharge and 45 days after LAAC using standardized interviews, documentation from referring physicians, and hospital discharge summaries. Each participant's demographic data were collected, including the modified Rankin scale, laboratory data, and imaging assessments, such as TTE and/or TEE. Device, technical, and procedural success were predefined as device deployed and implanted in correct position at the index procedure, exclusion of the LAA with site-reported residual jet ≤5 mm and no device-related complications through discharge or 7 days, whichever is earlier, and technical success with no procedure-related complications, respectively.^13^ For in-hospital data, all suspected serious adverse events, such as procedure-related complications (pericardial effusion, device embolization, access-related, TEE-related, atrial septal defect requiring closure, acute kidney injury, and surgical conversion), all-cause death, any stroke, and any bleeding were recorded. For follow-up data, all-cause death, device-related thrombus, all hospitalizations including stroke, bleeding, pericardial effusion, device embolization, LAA reintervention, infective endocarditis, surgical LAA occlusion, and worsened heart failure were collected for 45 days. Outcomes were independently adjudicated by the local clinical events committee of each center, according to the previous literature.^13^

### Prespecified definitions for age-related outcomes

All subjects were allocated into younger, middle-aged, and elderly groups according to age, as follows: 1) ≤70 years of age; 2) 70 to 80 years of age; and 3) >80 years of age, respectively. Collected data were compared among the 3 groups.

### Statistical analysis

Continuous variables are presented as mean ± SD or medians with interquartile ranges, as appropriate, whereas categorical variables are presented as frequencies with percentages. Groups were compared by one-way analysis of variance, Mann–Whitney U tests, or chi square tests as appropriate. The 2-tailed significance level was set at α < 0.05. Statistical analyses were performed using the software R x64 4.1.2 for Windows.

## Results

### Study population

The study included 548 patients from the registry who underwent LAAC between September 2019 and June 2021. The younger group included 104 patients (mean age, 64.1 ± 6.9 years; male, 76.9%), the middle-aged group included 271 patients (mean age, 75.9 ± 2.9 years; male, 73.1%), and the elderly group included 173 patients (mean age, 84.5 ± 2.6 years; male, 61.8%).

**Figure 1:**
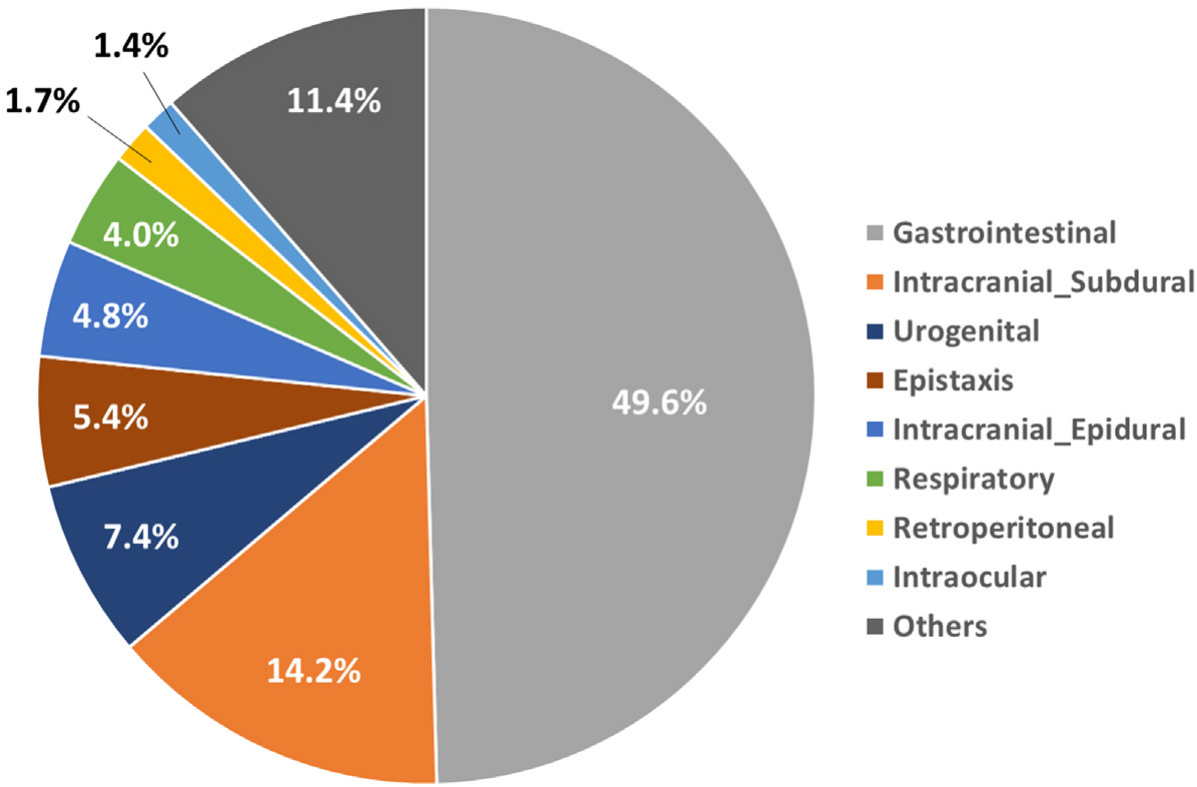
History of Bleeding Pie chart by preoperative bleeding history.

Baseline characteristics of the study population are shown in Table 1. Younger patients had higher body mass index and higher diastolic blood pressure compared with the body mass indices and diastolic pressures in the middle-aged and elderly groups. In addition, younger patients had a more frequent history of catheter ablation, cardioversion, any stroke/transient ischemic attack, and thrombotic events. Renal failure was more common in elderly patients (84.4%) compared with renal failure in younger (67.3%) and middle-aged (69.7%) patients (*P* = 0.001). Conversely, hemodialysis was more common in younger patients (20.2%) compared with the rate of hemodialysis in the elderly (6.4%) and middle-aged (13.3%) patients (*P* = 0.003). Baseline laboratory data and transthoracic echocardiography assessments are shown in Supplemental Table 2. Anemia, thrombocytopenia, and chronic kidney injury assessed by estimated glomerular filtration rate tended to be more common in the elderly group. Elderly patients had a higher tricuspid regurgitation pressure gradient, smaller left ventricular diastolic and systolic diameters, and more pronounced valvular dysfunction including mitral and tricuspid regurgitation compared with these cardiac parameters in younger and middle-aged patients. Of note, patients in the younger age group had a lower risk of falls, lower clinical frailty scale, lower HAS-BLED scores, and lower CHADS_2_ and CHA_2_DS_2_-VASc scores compared with the other 2 groups (Supplemental Table 3). The history of bleeding events is shown in Figure 1 and summarized in Supplemental Table 4. Gastrointestinal bleeding was the most frequently observed bleeding event and was significantly higher in the elderly group compared with bleeding in the other 2 groups.

**Table 1:** Baseline Characteristics

		Age, y			
	Overall(N = 548)	≤70(n = 104)	70 to 80(n = 271)	>80(n = 173)	*P* Value
Age, y	76.4 ± 8.1	64.1 ± 6.9	75.9 ± 2.9	84.5 ± 2.6	**<0.001**
Male	385 (70.3)	80 (76.9)	198 (73.1)	107 (61.8)	**0.01**
Body mass index, kg/m^2^	23.5 ± 3.9	25.2 ± 4.8	23.4 ± 3.6	22.8 ± 3.6	**<0.001**
Systolic blood pressure, mm Hg	125.5 ± 18.5	124.6 ± 18.0	126.7 ± 18.3	124.1 ± 19.2	0.32
Diastolic blood pressure, mm Hg	72.1 ± 13.3	75.8 ± 13.0	71.5 ± 12.7	67.6 ± 13.6	**<0.001**
Heart rate, beat/min	72.9 ± 14.6	74.4 ± 13.0	72.9 ± 14.5	72.1 ± 15.7	0.44
Rhythm					0.32
Sinus rhythm	168 (30.7)	37 (35.6)	86 (31.7)	45 (26.0)	
AF/AFL/AT	342 (62.4)	63 (60.6)	166 (61.3)	113 (65.3)	
Pacemaker rhythm	38 (6.9)	4 (3.8)	19 (7.0)	15 (8.7)	
Congestive heart failure	286 (52.2)	39 (37.5)	137 (50.6)	110 (63.6)	**<0.001**
NYHA functional class III or IV	17 (3.1)	1 (1.0)	6 (2.2)	10 (5.8)	**0.04**
Modified Rankin scale					0.12
No symptoms at all	384 (70.1)	69 (66.3)	183 (67.5)	132 (76.3)	
No significant disability despite symptoms	88 (16.1)	18 (17.3)	48 (17.7)	22 (12.7)	
Slight disability	37 (6.8)	7 (6.7)	19 (7.0)	11 (6.4)	
Moderate disability	25 (4.6)	8 (7.7)	13 (4.8)	4 (2.3)	
Moderately severe disability	6 (1.1)	1 (1.0)	5 (1.8)	0 (0.0)	
Severe disability	1 (0.2)	1 (1.0)	0 (0.0)	0 (0.0)	
Past medical history					
MI	99 (18.1)	15 (14.4)	47 (17.3)	37 (21.4)	0.31
PCI	182 (33.2)	25 (24.0)	91 (33.6)	66 (38.2)	0.053
CABG	37 (6.8)	5 (4.8)	17 (6.3)	15 (8.7)	0.42
Catheter ablation	106 (19.3)	33 (31.7)	57 (21.0)	16 (9.2)	**<0.001**
Surgical ablation	6 (1.1)	0 (0.0)	2 (0.7)	4 (2.3)	0.15
Cardioversion	21 (3.8)	7 (6.9)	12 (4.4)	2 (1.2)	**0.046**
Major bleeding	329 (60.0)	56 (53.8)	168 (62.0)	105 (60.7)	0.25
Any strokes/TIA	227 (41.4)	51 (49.0)	119 (43.9)	57 (32.9)	**0.016**
Thromboembolic event	167 (30.5)	44 (42.3)	84 (31.0)	39 (22.7)	**0.003**
Comorbidities					
Peripheral vascular disease	78 (14.2)	8 (7.7)	44 (16.2)	26 (15.0)	0.10
Chronic obstructive pulmonary disease	19 (3.5)	0 (0.0)	13 (4.8)	6 (3.5)	0.08
Carotid stenosis	27 (4.9)	3 (2.9)	16 (5.9)	8 (4.7)	0.47
Renal failure (eGFR <60 mL/min/1.73 m^2^)	405 (73.9)	70 (67.3)	189 (69.7)	146 (84.4)	**0.001**
Hemodialysis	68 (12.4)	21 (20.2)	36 (13.3)	11 (6.4)	**0.003**
Any cancers	110 (20.0)	14 (13.5)	58 (21.4)	38 (22.0)	0.19

Values are mean ± SD or n (%). **Bold** indicates *P* value <0.05.

AF = atrial fibrillation; AFL = atrial flutter; AT = atrial tachycardia; CABG = coronary artery bypass grafting; eGFR = estimated glomerular filtration rate; MI = myocardial infarction; NYHA = New York Heart Association; PCI = percutaneous coronary intervention; TIA = transient ischemic attack.

Preprocedural transesophageal echocardiographic assessments were similar among the 3 groups, except for the 3D major and minor axis of the LAA ostium diameter and the proportion of mitral annular calcification (Table 2).

**Table 2:** Preprocedural Transesophageal Echocardiography

		Age, y			
	Overall(N = 548)	≤70(n = 104)	70 to 80(n = 271)	>80(n = 173)	*P* Value
LAA ostium diameter, mm					
0°	21.71 ± 3.98	21.86 ± 3.69	21.74 ± 4.02	21.57 ± 4.12	0.87
45°	19.97 ± 3.54	19.81 ± 3.62	19.86 ± 3.37	20.26 ± 3.77	0.53
90°	20.62 ± 3.98	20.51 ± 3.86	20.57 ± 3.80	20.78 ± 4.34	0.86
135°	22.48 ± 4.28	22.83 ± 4.04	22.37 ± 4.07	22.42 ± 4.74	0.70
LAA depth, mm					
0°	28.68 ± 7.62	28.14 ± 7.96	28.53 ± 7.80	29.24 ± 7.11	0.58
45°	28.18 ± 7.01	27.97 ± 6.85	27.98 ± 6.94	28.62 ± 7.24	0.70
90°	28.61 ± 7.21	28.52 ± 7.37	28.60 ± 7.53	28.68 ± 6.62	0.99
135°	27.53 ± 6.85	28.52 ± 7.66	27.28 ± 6.23	27.33 ± 7.27	0.37
LAA ostium diameter (3D area), mm^2^	383.1 ± 132.3	411.1 ± 130.2	381.9 ± 130.1	373.5 ± 138.8	0.63
LAA ostium diameter (3D major axis), mm	23.84 ± 3.80	24.80 ± 3.19	24.07 ± 3.69	22.87 ± 4.17	**0.04**
LAA ostium diameter (3D minor axis), mm	19.46 ± 3.79	20.69 ± 3.40	19.57 ± 3.76	18.48 ± 3.90	**0.03**
LAA morphology					0.58
Windsock	125 (29.3)	28 (34.1)	62 (29.1)	35 (26.5)	
Cactus	96 (22.5)	17 (20.7)	47 (22.1)	32 (24.2)	
Cauliflower	115 (26.9)	20 (24.4)	64 (30.0)	31 (23.5)	
Chicken wing	91 (21.3)	17 (20.7)	40 (18.8)	34 (25.8)	
Spontaneous echo contrast grade					0.89
1	133 (30.7)	24 (29.3)	66 (30.6)	43 (31.9)	
2	71 (16.4)	13 (15.9)	40 (18.5)	18 (13.3)	
3	42 (9.7)	7 (8.5)	19 (8.8)	16 (11.9)	
4	11 (2.5)	1 (1.2)	6 (2.8)	4 (3.0)	
LAA thrombus	3 (0.7)	1 (1.2)	1 (0.5)	1 (0.7)	0.79
LAA filling flow velocity, m/s	31.6 ± 19.1	31.3 ± 16.5	33.1 ± 19.8	29.1 ± 19.5	0.25
LAA emptying flow velocity, m/s	31.1 ± 20.2	33.0 ± 20.7	32.4 ± 20.3	27.8 ± 19.3	0.15
Pericardial effusion	13 (3.0)	4 (4.8)	6 (2.8)	3 (2.2)	0.53
MAC	28 (6.5)	1 (1.2)	8 (3.7)	19 (14.1)	**<0.001**

Values are mean ± SD or n (%). **Bold** indicates *P* value <0.05.

LAA = Left atrial appendage; MAC = Mitral annular calcification.

### Procedural outcomes

Procedural variables and key clinical endpoints are summarized in Tables 3 and 4. Procedural characteristics were similar in the 3 groups; however, red blood cell transfusions were more common in the elderly group. A double-curve system was selected for device implantation in two-thirds of the cases. The average number of access system use and device use in all procedures was 1.04 ± 0.21 and 1.24 ± 0.57. Septal puncture locations were changed in eight patients (1.5%) for optimal device implantation.

**Table 3:** Procedural Characteristics

		Age, y			
	Overall(N = 548)	≤70(n = 104)	70 to 80(n = 271)	>80(n = 173)	*P* Value
Anesthesia time, min	101.0 (82.0-125.2)	102.5 (84.8-123.5)	100.0 (83.0-123.0)	103.0 (80.0-132.0)	0.69
Procedural time, min	54.0 (40.0-72.0)	53.5 (40.8-68.3)	53.0 (40.0-71.0)	56.0 (40.0-79.0)	0.41
Contrast volume, mL	68.3 ± 44.5	69.7 ± 37.1	71.8 ± 51.3	62.0 ± 35.9	0.07
Fluoroscopy duration, min	12.4 (8.8-17.0)	11.0 (8.8-17.9)	12.4 (8.1-16.0)	13.0 (9.0-18.4)	0.08
Concomitant procedure	14 (2.6)	3 (2.9)	4 (1.5)	7 (4.0)	0.24
RBC transfusion	11 (2.0)	1 (1.0)	3 (1.1)	7 (4.0)	0.07
Preprocedural mean left atrial pressure, mm Hg	13.0 ± 4.3	13.3 ± 4.6	12.7 ± 3.7	13.2 ± 4.8	0.30
Type of access system at deployment					0.98
Single curve	178 (32.9)	33 (32.0)	89 (33.2)	56 (32.9)	
Double curve	363 (67.1)	70 (67.3)	179 (66.1)	114 (65.9)	
Anterior curve	0 (0.0)	0 (0.0)	0 (0.0)	0 (0.0)	
Type of implanted device					0.35
WATCHMAN 2.5	504 (94.6)	99 (96.1)	248 (94.3)	157 (94.06)	
21-mm	19 (3.6)	3 (2.9)	11 (4.2)	5 (3.0)	
24-mm	56 (10.5)	14 (13.6)	22 (8.4)	20 (12.0)	
27-mm	124 (23.3)	24 (23.3)	52 (19.8)	48 (28.7)	
30-mm	123 (23.1)	28 (27.2)	62 (23.6)	33 (19.8)	
33-mm	182 (34.1)	30 (29.1)	101 (38.4)	51 (30.5)	
WATCHMAN FLX	29 (5.4)	4 (3.9)	15 (5.7)	10 (6.0)	
20-mm	1 (0.2)	1 (1.0)	0 (0.0)	0 (0.0)	
24-mm	2 (0.4)	0 (0.0)	2 (0.8)	0 (0.0)	
27-mm	7 (1.3)	0 (0.0)	4 (1.5)	3 (1.8)	
31-mm	9 (1.7)	2 (1.9)	4 (1.5)	3 (1.8)	
35-mm	10 (1.9)	1 (1.0)	5 (1.9)	4 (2.4)	
No. of access system use	1.04 ± 0.21	1.06 ± 0.31	1.03 ± 0.16	1.05 ± 0.21	0.36
No. of device use	1.24 ± 0.57	1.18 ± 0.50	1.26 ± 0.60	1.24 ± 0.56	0.51
Change of septal puncture location	8 (1.5)	2 (2.0)	4 (1.5)	2 (1.2)	0.87

Values are median (IQR), n (%), or mean ± SD.

RBC = red blood cells.

**Table 4:** Key Procedural Endpoints

		Age, y			
	Overall(N = 548)	≤70(n = 104)	70 to 80(n = 271)	>80(n = 173)	*P* Value
Device success^a^	529 (96.5)	103 (99.0)	261 (96.3)	165 (95.4)	0.26
Technical success^b^	526 (96.0)	103 (99.0)	260 (95.9)	163 (94.2)	0.14
Procedural success^c^	496 (90.5)	97 (93.3)	251 (92.6)	148 (85.5)	**0.03**
TEE assessment after device implantation, mm					
Device compression 0°	17.7 ± 7.2	17.5 ± 6.6	17.7 ± 7.1	17.9 ± 7.8	0.90
Device compression 45°	18.1 ± 6.5	17.8 ± 6.4	18.6 ± 6.7	17.4 ± 6.3	0.17
Device compression 90°	17.6 ± 6.7	17.9 ± 6.9	17.8 ± 6.8	17.3 ± 6.3	0.73
Device compression 135°	16.6 ± 6.6	16.8 ± 6.4	16.2 ± 6.3	17.3 ± 7.0	0.20
Peridevice leak, mm					**0.001**
0	492 (92.7)	87 (84.5)	244 (93.1)	161 (97.0)	
<3	39 (7.3)	16 (15.5)	18 (6.9)	5 (3.0)	
3-5	0 (0.0)	0 (0.0)	0 (0.0)	0 (0.0)	
≥5	0 (0.0)	0 (0.0)	0 (0.0)	0 (0.0)	
Right-left shunt of ASD flow	65 (16.6)	9 (11.8)	37 (18.4)	19 (16.4)	0.42
Post spontaneous echo contrast, grade					0.48
1	129 (24.1)	32 (31.4)	59 (22.3)	38 (22.6)	
2	62 (11.6)	8 (7.8)	34 (12.8)	20 (11.9)	
3	34 (6.4)	4 (3.9)	20 (7.5)	10 (6.0)	
4	9 (1.7)	3 (2.9)	3 (1.1)	3 (1.8)	
Deep device implantation	15 (2.8)	5 (4.9)	7 (2.7)	3 (1.8)	0.34
Residual trabeculation	36 (6.8)	12 (11.8)	18 (6.8)	6 (3.6)	**0.04**
Length of hospital stay, days	5.0 (4.0-6.0)	4.0 (4.0-6.0)	4.0 (4.0-6.0)	5.0 (4.0-8.0)	**0.02**
Length of ICU/CCU stay, days	0.0 (0.0-1.0)	0.0 (0.0-1.0)	0.0 (0.0-1.0)	0.0 (0.0-1.0)	0.31
Discharge destination					0.63
Home (alone)	140 (25.5)	28 (26.9)	63 (23.2)	49 (28.3)	
Home (with family)	391 (71.2)	73 (70.2)	201 (74.2)	117 (67.6)	
Hospital or nursing home	17 (3.1)	3 (2.9)	7 (2.6)	7 (4.0)	

Values are median (IQR), n (%), or mean ± SD. **Bold** indicates P value <0.05.

ASD = atrial septal defect; CCU = cardiac care unit; ICU = intensive care unit; TEE = transesophageal echocardiography.

a Defined as device deployed and implanted in correct position at the index procedure.

b Exclusion of the LAA with site-reported residual jet ≤5 mm and no device-related complications through discharge or 7 days, whichever is earlier.

c Technical success with no procedure-related complications.

Overall, device implantation was successful in 529 patients (96.5%). A large LAAC device or new-generation LAAC device larger than 30 mm was implanted in 324 (60.8%) patients. In addition, technical and procedural success were achieved in 96.0% and 90.5% of patients, respectively. TEE assessments after device implantation revealed more peridevice leaks (PDLs) in the younger group, resulting in a higher frequency of residual trabeculation. However, no PDLs larger than 3 mm occurred immediately after device implantation in all cases. Hospital stays were significantly longer in the elderly group than those in the other 2 groups (*P* = 0.02). The comparison between preprocedural and intraoperative (before device deployment) TEE assessments is shown in Supplemental Table 5. Interestingly, although significant differences in LAA depth between the 2 measurement timepoints were detected across all ages, no differences in the LAA ostium diameter used to determine device size were detected.

### Periprocedural drug regimen

**Figure 2:**
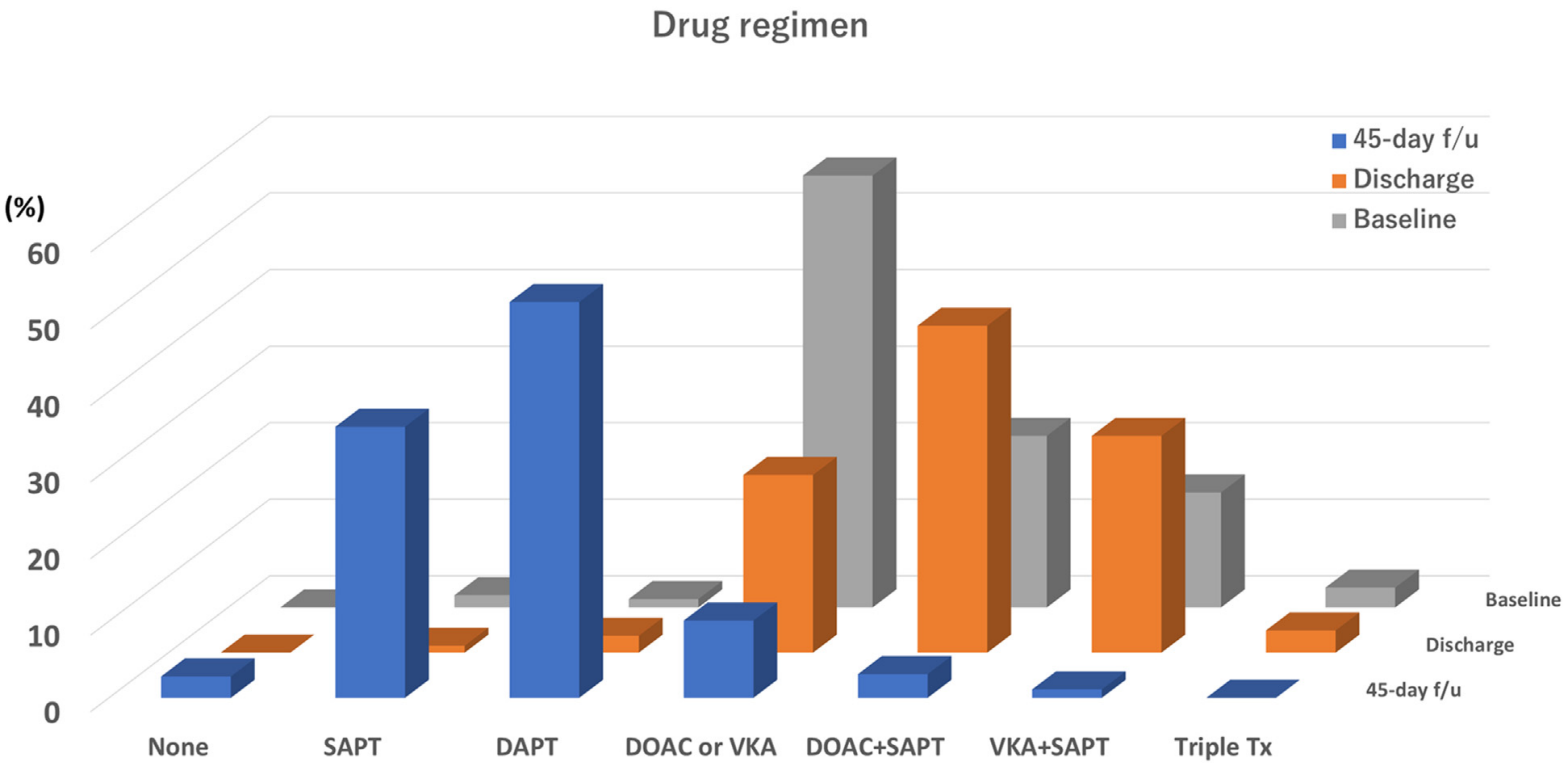
Periprocedural Drug Regimens Drug regimens at admission, discharge, and the 45-day follow-up (f/u) are shown as 3-dimensional bar charts. DAPT = dual antiplatelet therapy; DOAC = direct oral anticoagulant; SAPT = single antiplatelet therapy; Tx = therapy; VKA = vitamin K antagonist.

Changes in the drug regimen of the present cohort are shown in Figure 2, and details according to age are shown in Supplemental Table 6. At baseline, a total of 528 (96.4%) patients were on anticoagulants, and 532 (97.1%) patients were on anticoagulants postoperatively. In Japanese DOAC users, apixaban and edoxaban were favorably selected and edoxaban was used significantly more in the elderly group. At the 45-day follow-up, anticoagulants were discontinued in 89.9% of the cases and the majority were switched to either dual- or single-antiplatelet therapy.

### TEE assessments at the 45-day follow-up

Clinical follow-up data at 45 days after LAAC were collected from 527 (96.2%) patients, including 444 (84.3%) patients who underwent TEE for imaging assessments of the LAAC device. Detailed TEE assessments were similar among the age groups, as shown in Supplemental Table 7. No PDLs were observed at any angles in 292 (65.8%) patients. However, 3- to 5-mm PDLs were found in 38 (8.5%) patients, and larger PDLs of more than 5 mm were detected in only 2 (0.5%) patients. In addition, deep device implantation, which was predefined as when the device was implanted more than 10 mm from the tip of the coumadin ridge, was observed in 14 (3.2%) patients and device-related thrombus (DRT) was detected in 6 (1.4%) patients with no significant differences among groups.

### Clinical outcomes up to the 45-day follow-up

Event rates for clinical outcomes in the hospital and during the 45-day follow-up are provided in Table 5. During the procedure, surgical conversion was observed in 3 patients due to the LAA perforated by a sheath resulting in cardiac tamponade with circulatory collapse. With regards to in-hospital events, elderly patients had a numerically higher risk of any bleeding compared with the bleeding risk in the younger and middle-aged groups (younger vs middle-aged vs elderly, 1.9% vs 1.5% vs 4.6%, respectively; *P* = 0.11). However, all-cause death, any stroke events, pericardial effusion, device embolization, and procedure-related complications were similar among the groups.

**Table 5:** Clinical Outcomes Up to 45 Days Follow-Up

		Age, y			
	Overall(N = 548)	≤70(n = 104)	70 to 80(n = 271)	>80(n = 173)	*P* Value
In-hospital					
All-cause death	0 (0.0)	0 (0.0)	0 (0.0)	0 (0.0)	NA
Cardiovascular death	0 (0.0)	0 (0.0)	0 (0.0)	0 (0.0)	NA
Any stroke events	0 (0.0)	0 (0.0)	0 (0.0)	0 (0.0)	NA
Any bleeding events	14 (2.6)	2 (1.9)	4 (1.5)	8 (4.6)	0.11
Minor^a^	7 (1.3)	2 (1.9)	1 (0.4)	4 (2.3)	0.17
Major^b^	7 (1.3)	0 (0.0)	3 (1.1)	4 (2.3)	0.24
Pericardial effusion	11 (2.0)	1 (1.0)	4 (1.5)	6 (3.5)	0.24
Device embolization	0 (0.0)	0 (0.0)	0 (0.0)	0 (0.0)	NA
Surgical conversion	3 (0.5)	0 (0.0)	1 (0.4)	2 (1.2)	0.39
Access site-related complications	5 (0.9)	2 (1.9)	1 (0.4)	1 (0.6)	0.34
TEE-associated complications	7 (1.3)	0 (0.0)	4 (1.5)	2 (1.2)	0.42
Acute kidney injury	5 (0.9)	0 (0.0)	1 (0.4)	4 (2.3)	0.06
ASD requiring closure	1 (0.2)	1 (1.0)	0 (0.0)	0 (0.0)	0.12
At 45 days					
All-cause death	2 (0.4)	1 (1.0)	1 (0.4)	0 (0.0)	0.44
Cardiovascular death	0 (0.0)	0 (0.0)	0 (0.0)	0 (0.0)	NA
All-hospitalization	29 (5.3)	4 (3.8)	15 (5.5)	10 (5.8)	0.76
Any stroke events	2 (0.4)	0 (0.0)	2 (0.7)	0 (0.0)	0.36
Any bleeding events	40 (7.3)	6 (5.8)	16 (5.9)	18 (10.4)	0.17
Minor^a^	17 (3.1)	5 (4.8)	6 (2.2)	6 (3.5)	0.41
Major^b^	23 (4.2)	1 (1.0)	10 (3.7)	12 (6.9)	**0.047**
Pericardial effusion	13 (2.4)	1 (1.0)	4 (1.5)	8 (4.6)	0.06
Device embolization	0 (0.0)	0 (0.0)	0 (0.0)	0 (0.0)	NA
DRT	6 (1.4)	1 (1.2)	4 (1.8)	1 (0.7)	0.68
LAAC reintervention	0 (0.0)	0 (0.0)	0 (0.0)	0 (0.0)	NA
Surgical LAA occlusion	0 (0.0)	0 (0.0)	0 (0.0)	0 (0.0)	NA
Infective endocarditis	0 (0.0)	0 (0.0)	0 (0.0)	0 (0.0)	NA

Values are n (%). **Bold** indicates *P* value <0.05.

DRT = device-related thrombus; LAAC = left atrial appendage closure; NA = not applicable; other abbreviations as in Tables 2 and 4.

a Minor bleeding was defined as BARC type 2.

b Major bleeding was defined as BARC type 3a or greater.

**Figure 3:**
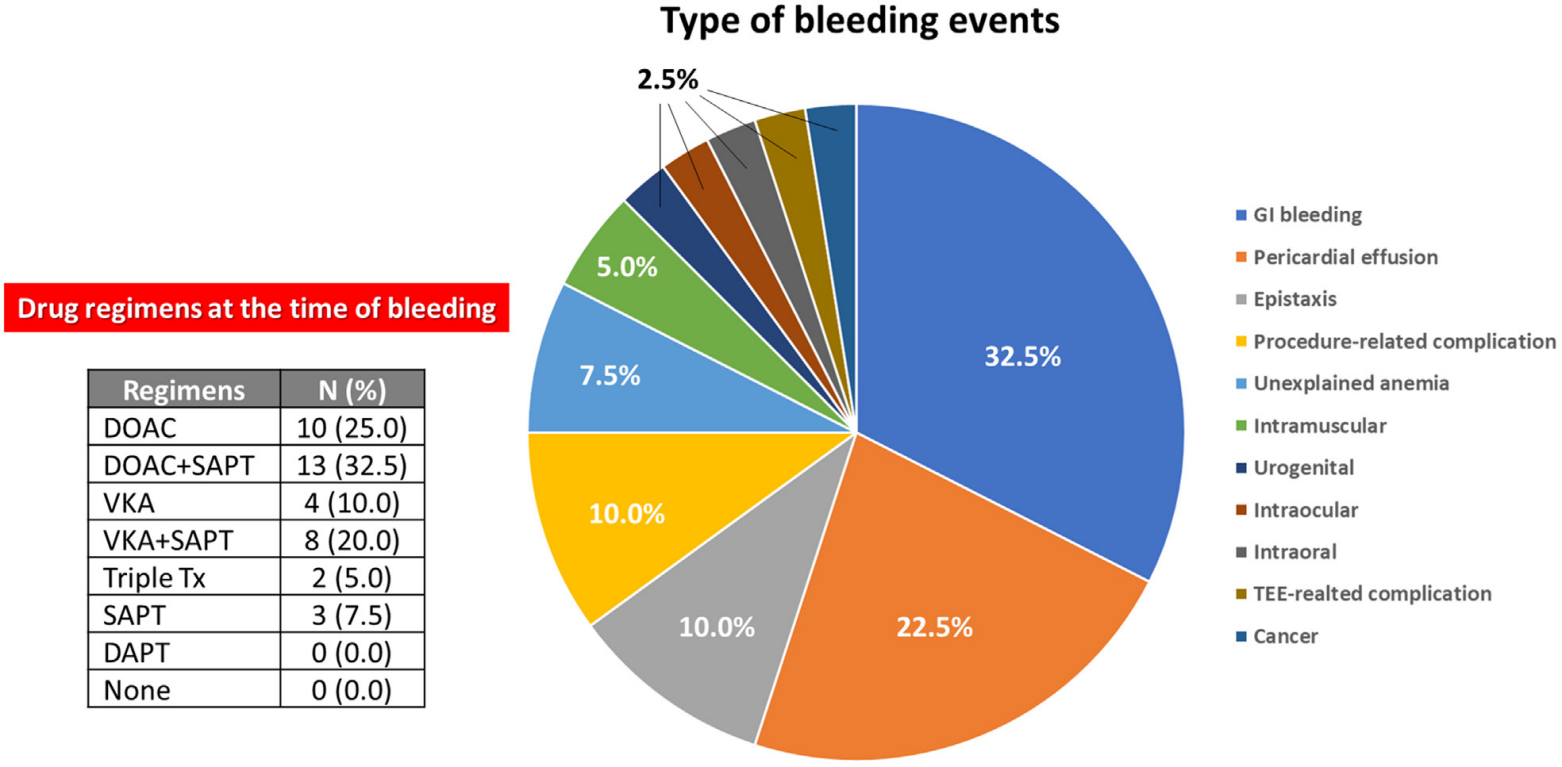
Bleeding Events and Drug Regimens at the Time of Bleeding **(Left)** Drug regimens at the time of bleeding events. **(Right)** Pie chart for detailed type of postoperative bleeding events. GI = gastrointestinal; TEE = transesophageal echocardiography; other abbreviations as in Figure 2.

During the 45-day follow-up, any bleeding events were noted in 40 (7.3%) patients, and elderly patients had a significantly higher incidence of bleeding events than the other groups (1.0% vs 3.7% vs 6.9%; *P* = 0.047). Detailed bleeding events are depicted in Figure 3. Gastrointestinal bleeding was observed in one-third, followed by pericardial effusion, epistaxis, procedure-related complications, unexplained anemia, and intramuscular hemorrhage (Supplemental Table 8). The drug regimens at the time of bleeding events have been selected a combination therapy of anticoagulants and antiplatelet agents in two-thirds (Supplemental Table 9). Furthermore, pericardial effusion rates were higher in elderly patients compared to the other 2 groups, but the difference did not reach statistical significance (1.0% vs 1.5% vs 4.6%; *P* = 0.06). All-cause death was documented in only 2 individuals (0.4%). Both died of noncardiac causes, one in a traffic accident, the other of lung cancer. No significant differences in all hospitalizations, and stroke events were detected among groups. Notably, late pericardial effusion was found in 2 patients within 45 days after LAAC. One was diagnosed with drug-induced pericardial effusion and the other was diagnosed with post-cardiac injury syndrome. Both had no cardiac tamponade, but pericardiocentesis had been performed to treat it and to confirm the nature of pericardial fluid; it was not bloody. No device embolization, LAAC reintervention, surgical LAA occlusion, or infective endocarditis occurred.

## Discussion

The present analysis of patients from a large prospective multicenter registry shows the initial Japanese experience in patients undergoing percutaneous LAAC according to age. There are several key findings from this study (Central Illustration). LAAC with the LAAC devices was successful in 96.5% of patients with a mean CHADS_2_ score of 3.1 ± 1.3, CHA_2_DS_2_-VASc score of 4.7 ± 1.5, and HAS-BLED score of 3.2 ± 1.0 points. The procedure success, defined as no procedure-related complications, was 90.5% due to the high incidence of postoperative bleeding in the elderly population. At the 45-day follow-up, anticoagulants cessation was achieved in 89.9% of the cases. Deaths from any causes were documented in only 2 (0.4%); both were noncardiac. A few stroke events (0.4%) and DRT (1.4%) were observed with no differences according to age. In contrast, bleeding (7.3%) and pericardial effusion (2.4%) tended to be more common in the elderly group.

**Central Illustration:**
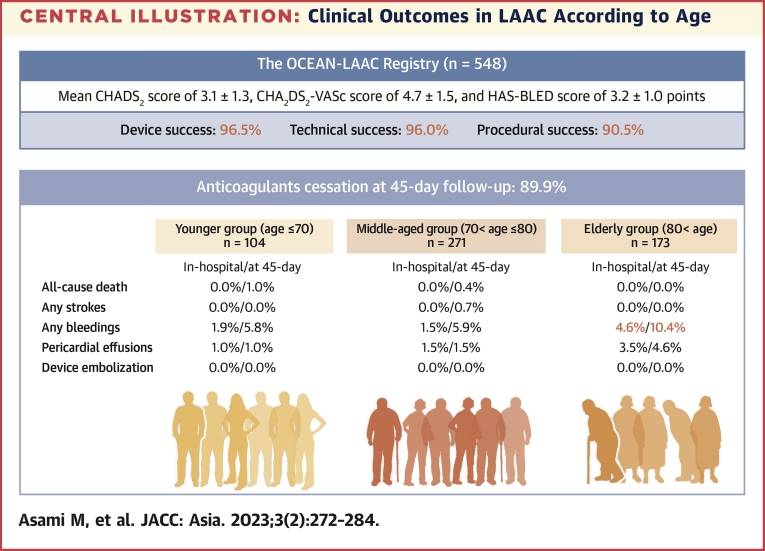
Clinical Outcomes in LAAC According to Age **(Upper)** Overall background of the study population and procedural outcomes of left atrial appendage closure (LAAC) in the OCEAN-LAAC registry. **(Lower)** In-hospital and short-term clinical outcomes according to age.

Limited multicenter studies of LAAC in Asians have been conducted, with none in the Japanese population. In the Registry on WATCHMAN Outcomes in Real-Life Utilization study, which included 107 Asians and 94 non-Asians from 7 countries, the Asian participants (mean age: 70.7 ± 9.4 years, 62.6% male) had a mean CHADS_2_ score of 2.5 ± 1.3, a mean CHA_2_DS_2_-VASc score of 4.1 ± 1.7, and a mean HAS-BLED score of 2.2 ± 1.3.^14^ In this setting, the device success was 99.1%, bleeding events were observed in 1% of patients within 30 days, and stroke occurred in only 1 patient. Furthermore, 3,096 patients (mean age of 69.1 ± 9.4 years, 57.6% male) with a mean CHA_2_DS_2_-VASc score of 4.0 ± 1.8 and a mean HAS-BLED score of 2.4 ± 1.2 were included in a Chinese multicenter registry.^15^ The device success was 99.5%, stroke events occurred in 9 patients, major bleeding occurred in 33 patients, and minor bleeding occurred in 14 patients within 30 days. Initial Japanese data were reported in the SALUTE (A Study to Evaluate the Safety and Effectiveness of the Left Atrial Appendage Closure Therapy Using BSJ003W for Patients With Non-valvular Atrial Fibrillation at Increased Risk of ThromboEmbolism in Japanese Medical Environment) trial, which was a small, prospective, single-arm registry of 54 participants (mean age of 72.5 ± 8.8 years, 83.3% male), with a mean CHADS_2_ score of 2.5 ± 1.3, a mean CHA_2_DS_2_-VASc score of 3.6 ± 1.6, and a mean HAS-BLED score of 2.9 ± 1.1.^16^ Unlike previous reports, approximately 90% of the subjects had bleeding events and/or high bleeding tendency. Device success was 100%, whereas perioperative all-cause mortality or stroke was 0%, major bleeding was 0%, and minor bleeding was noted in only 1 individual.

The mean age of the participants was older (76.4 ± 8.1 years), and the CHADS_2_ scores (3.1 ± 1.3), CHA_2_DS_2_-VASc scores (4.7 ± 1.5), and HAS-BLED scores (3.2 ± 1.0) were higher in the current study than in the previous reports.^17^, ^18^, ^19^, ^20^ This is the first real-world multicenter registry of LAAC in Japan, and the results are comparable to previous reports despite having an increased risk of both bleeding and thromboembolism.^8^^,^^10^^,^^21^ Notably, the results of the present study in the younger age group were extremely favorable. Interpreting the results by age, the elderly group had a higher clinical frailty scale and was at an increased risk of falls. Furthermore, as age increased, CHADS_2_ and CHA_2_DS_2_-VASc scores trended upward due to the increased frequency of heart failure and vascular complications. Meanwhile, the HAS-BLED score, which indicates bleeding risk, was high for all ages. However, a detailed breakdown of the scores showed that younger patients had a more frequent history of stroke, while elderly patients exhibited more drug abuse.

A large retrospective study enrolling 6,877 patients compared clinical outcomes of LAAC between 2 groups dichotomizing at 75 years old. Higher incidences of cardiac perforation and cardiac tamponade were observed in older females.^22^ A subanalysis of the EWOLUTION (Evaluating Real-Life Clinical Outcomes in Atrial Fibrillation Patients Receiving the Watchman Left Atrial Appendage Closure Technology) registry, which is a European real-world registry including 1,025 participants, compared clinical outcomes between 2 groups divided at 85 years of age. The study showed that LAAC can be safe and effective in patients older than 85 years of age despite the higher risk of embolism and bleeding.^23^ In addition, a prospective study using Amulet involving 1,088 patients divided into 3 age groups similar to the present study reported no differences in procedure-related complications and a successful reduction in the incidence of ischemic stroke in all ages after the intervention despite an increased risk of embolic events with increasing age.^24^ The differences between these previous studies and ours can be attributed to differences in the experience with LAAC and a higher-risk of baseline characteristics, including the increased age and risk scores for bleeding and embolism.

In our study, preoperative and intraoperative TEE measurements were compared by age. Remarkably, the ostium diameter of LAA measured by preoperative TEE with saline loading before the examination similar to a previously reported method was comparable to the intraoperative ostium diameter, indicating that the device size was correctly predicted by preoperative TEE in all age groups.^25^ In contrast, the depth of LAA tended to be shorter. The difference may be due to the results of measurements may have been taken at the deepest point of LAA, which cannot be implanted the device because of the inability to assume the optimal location of the measurements before the procedure by the imaging specialists in Japan from the lack of experience. Interestingly, the LAA size at baseline tended to be larger in younger patients, which may have been resulted from the fact that only NVAF patients with high bleeding risk were indicated for LAAC in Japan, and mainly particular populations, such as dialysis patients, were considered the eligible for the intervention in the younger group.

No significant differences in perioperative drug regimens were detected among the age groups, but edoxaban was preferentially chosen in the elderly group when selecting DOACs.^26^ However, the same postoperative drug regimen is recommended for all ages in Japan. The elderly group had a higher rate of anticoagulant discontinuation at the 45-day follow-up (94.2%); however, postoperative bleeding events occurred more frequently during combination therapy with anticoagulants and antiplatelet agents up to 45 days. Therefore, the postoperative drug regimen should be tailored for elderly patients, considering their background factors.

### Study limitations

The findings of the present analysis must be interpreted in light of several limitations. First, the follow-up period was limited. A 45-day follow-up is insufficient to evaluate the clinical outcomes of LAAC, which is an invasive treatment, although it is percutaneous. However, our registry adheres to high standards of data quality with rigorous data collection, regular follow-up, and event adjudication by experts. Second, in the present analyses of clinical endpoints, we cannot rule out residual confounding effects by variables not recorded in our database.

## Conclusions

The initial Japanese experience in a multicenter registry showed that LAAC was safe and effective. However, perioperative bleeding events were observed more frequently in the elderly; therefore, age-specific post-LAAC drug regimens should be considered.

Perspectives **COMPETENCY IN PRACTICE-BASED LEARNING:** Percutaneous LAAC is a safe and effective alternative to anticoagulants in patients with AF; however, few reports describe the use of LAAC in Asians, especially the Japanese. The initial experience in Japan was favorable, as in previous studies, but the age-specific analysis showed a higher incidence of adverse events in patients older than 80 years of age.

**TRANSLATIONAL OUTLOOK:** This study is the first report from a multicenter registry of LAAC in Japan. Our results support the safety and efficacy of LAAC and provide an opportunity to resurvey the postoperative drug regimen in elderly patients. Further larger and longer-term follow-up studies are warranted to demonstrate the usefulness of LAAC in the Asian population.

## Funding Support and Author Disclosures

The OCEAN-LAAC registry, which is part of OCEAN-SHD registry, is supported by Edwards Lifesciences, Medtronic, Boston Scientific, Abbott Medical, and Daiichi Sankyo Company. Drs Asami, Ueno, Kubo, and Yamamoto are clinical proctors for Boston Scientific. Dr Asami has received speaker fees from Daiichi Sankyo Company, Bristol Myers Squibb, Pfizer, Boehringer Ingelheim, and Boston Scientific. Dr Naganuma has received speaker fees from Daiichi Sankyo Company. Dr Ohno has received lecture fees from Daiichi Sankyo Company, Bristol Myers Squibb, and Boston Scientific. Dr Izumo has received speaker fees from Daiichi Sankyo Company, Bayer, and Bristol Myers Squibb. Dr Saji has received speaker fees from Abbott Medical. Dr Ueno has received speaker fees from Daiichi Sankyo Company, Bayer, Bristol Myers Squibb, Pfizer, Boehringer Ingelheim, and Boston Scientific. Dr Kubo has received speaker fees from Daiichi Sankyo Company and Boehringer Ingelheim. Dr Nakashima has received speaker fees from Daiichi Sankyo Company, Bristol Myers Squibb, and Boston Scientific. Dr Yamamoto has received speaker fees from Daiichi Sankyo Company, Bayer, Bristol Myers Squibb, and Pfizer. Dr Hayashida has received speaker fees from Daiichi Sankyo Company, Bayer, Bristol Myers Squibb, and Pfizer. The remaining authors have reported that they have no relationships relevant to the contents of this paper to disclose.
