# Supplementary material for: Initial Japanese Multicenter Experience and Age-Related Outcomes Following Left Atrial Appendage Closure: The OCEAN-LAAC Registry
Source: JACC Asia. 2023 Feb 14;3(2):272–84. doi: 10.1016/j.jacasi.2022.11.003 (PMC10167511; doi:10.1016/j.jacasi.2022.11.003)
Supplement: Supplemental Tables 1–9 [file mmc1.docx]

**Supplemental Table 1 Participating Sites and Investigators**

| **Site** | **Department** | **Investigator** | **Role** | **Location** |
| --- | --- | --- | --- | --- |
| Keio University School of Medicine | Department of Cardiology | Kentaro Hayashida | Site PI | Tokyo |
| Toyohashi Heart Center | Department of Cardiology | Masanori Yamamoto | Site PI | Aichi |
| Nagoya Heart Center | Department of Cardiology | Masanori Yamamoto | Site PI | Aichi |
| Gifu Heart Center | Department of Cardiology | Masanori Yamamoto | Site PI | Gifu |
| Kokura Memorial Hospital | Department of Cardiology | Shinichi Shirai | Site PI | Fukuoka |
| Teikyo University School of Medicine | Department of Cardiology | Yusuke Watanabe | Site PI | Tokyo |
| New Tokyo Hospital | Department of Cardiology | Toru Naganuma | Site PI | Chiba |
| Sendai Kousei Hospital | Department of Cardiology | Masaki Nakashima | Site PI | Miyagi |
| Shonan Kamakura General Hospital | Department of Cardiology | Shingo Mizuno | Site PI | Kanagawa |
| Kinki University school of Medicine | Department of Cardiology | Kazuki Mizutani | Site PI | Osaka |
| Toyama University Hospital | Department of Cardiology | Hiroshi Ueno | Site PI | Toyama |
| Tokai University School of Medicine | Department of Cardiology | Yohei Ohno | Site PI | Kanagawa |
| St. Marianna University School of Medicine | Department of Cardiology | Masaki Izumo | Site PI | Kanagawa |
| Sapporo East Tokushukai Hospital | Department of Cardiology | Tomoyuki Tani | Site PI | Hokkaido |
| Saiseikai Kumamoto Hospital | Department of Cardiology | Hideharu Okamatsu | Site PI | Kumamoto |
| Kurashiki central Hospital | Department of Cardiology | Shunsuke Kubo | Site PI | Okayama |
| Mitsui Memorial Hospital | Division of Cardiology | Masahiko Asami | Site PI | Tokyo |
| Sakakibara Heart Institute | Department of Cardiology | Mike Saji | Site PI | Tokyo |

**Supplemental Table 2 Baseline laboratory data and transthoracic echocardiography assessments**

|  | **Overall** | **Age <=70** | **70< Age <=80** | **80< Age** | **p-value** |
| --- | --- | --- | --- | --- | --- |
|  | N = 548 | N = 104 | N = 271 | N = 173 |  |
| **Laboratory data** |  |  |  |  |  |
| Hemoglobin, g/dl | 12.1 ± 2.2 | 12.9 ± 2.3 | 12.3 ± 2.2 | 11.4 ± 1.8 | **<0.001** |
| Platelet, /μl | 18.9 ± 6.8 | 20.9 ± 7.0 | 19.1 ± 6.9 | 17.6 ± 6.2 | **<0.001** |
| Albumin, g/dl | 3.85 ± 0.46 | 3.92 ± 0.51 | 3.88 ± 0.46 | 3.75 ± 0.42 | **0.002** |
| Serum creatinine, mg/dl | 1.90 ± 2.10 | 2.27 ± 2.61 | 1.95 ± 2.27 | 1.59 ± 1.27 | **0.03** |
| eGFR, mL/min/1.73m^2^ | 43.7 ± 22.4 | 46.0 ± 25.2 | 45.2 ± 23.1 | 39.9 ± 18.7 | **0.03** |
| AST, U/L | 24.5 ± 14.5 | 23.1 ± 13.8 | 24.6 ± 11.8 | 25.1 ± 18.3 | 0.52 |
| ALT, U/L | 19.2 ± 18.3 | 22.4 ± 32.1 | 19.4 ± 12.1 | 16.9 ± 14.5 | **0.048** |
| BNP, pg/ml | 245.3 ± 290.1 | 215.8 ± 310.7 | 244.5 ± 287.1 | 267.1 ± 280.9 | 0.50 |
| NT-proBNP, pg/ml | 4211.0 ± 8094.2 | 5913.6 ± 10581.8 | 4299.2 ± 8525.3 | 3355.2 ± 5944.6 | 0.27 |
| PT-INR | 1.54 ± 0.53 | 1.51 ± 0.52 | 1.56 ± 0.55 | 1.51 ± 0.49 | 0.52 |
| D-Dimer, μg/ml | 1.57 ± 3.89 | 1.39 ± 3.05 | 1.59 ± 4.54 | 1.64 ± 3.20 | 0.92 |
| **Transthoracic echocardiography** |  |  |  |  |  |
| LVDd, mm | 48.4 ± 7.7 | 50.3 ± 7.2 | 48.6 ± 7.9 | 47.0 ± 7.5 | **0.002** |
| LVDs, mm | 33.1 ± 8.8 | 34.6 ± 8.9 | 33.3 ± 8.9 | 31.8 ± 8.5 | **0.04** |
| LAV Index, mL/m^2^ | 61.5 ± 28.9 | 56.5 ± 24.7 | 60.9 ± 29.4 | 66.0 ± 30.3 | 0.14 |
| LVEF, % | 57.7 ± 12.4 | 56.5 ± 12.3 | 57.3 ± 12.5 | 59.2 ± 12.2 | 0.17 |
| TRPG, mmHg | 27.6 ± 9.3 | 25.4 ± 8.8 | 27.1 ± 8.5 | 29.6 ± 10.4 | **0.001** |
| AR moderate or severe, n (%) | 23 (4.3) | 3 (2.9) | 10 (3.8) | 10 (5.9) | 0.42 |
| MR moderate or severe, n (%) | 73 (13.6) | 5 (4.9) | 35 (13.2) | 33 (19.5) | **0.003** |
| TR moderate or severe, n (%) | 98 (18.2) | 4 (3.9) | 51 (19.2) | 43 (25.4) | **<0.001** |

Counts with percentages (%) or means ± standard deviations are shown.

Abbreviations: AR = aortic regurgitation; eGFR = estimated glomerular filtration rate; LAV = Left atrial volume; LVDd = left ventricular diastolic diameter; LVDs = left ventricular systolic diameter; LVEF = left ventricular ejection fraction; MR = Mitral regurgitation; TR = Tricuspid regurgitation; TRPG = Tricuspid regurgitation pressure gradient.

**Supplemental Table 3 Risk Scores**

|  | **Overall** | **Age <=70** | **70< Age <=80** | **80< Age** | **p-value** |
| --- | --- | --- | --- | --- | --- |
|  | N = 548 | N = 104 | N = 271 | N = 173 |  |
| Increased risk of fall, n (%) | 146 (26.6) | 14 (13.5) | 62 (22.9) | 70 (40.5) | **<0.001** |
| Mean clinical frailty scale | 3.0 ± 1.3 | 2.5 ± 1.4 | 3.0 ± 1.2 | 3.5 ± 1.3 | **<0.001** |
| Clinical frailty scale |  |  |  |  | **<0.001** |
| Very Fit, n (%) | 69 (12.6) | 25 (24.0) | 29 (10.7) | 15 (8.7) |  |
| Fit, n (%) | 122 (22.3) | 34 (32.7) | 65 (24.0) | 23 (13.3) |  |
| Managing Well, n (%) | 177 (32.3) | 24 (23.1) | 105 (38.7) | 48 (27.7) |  |
| Living with Very Mild Frailty, n (%) | 110 (20.1) | 12 (11.5) | 43 (15.9) | 55 (31.8) |  |
| Living with Mild Frailty, n (%) | 48 (8.8) | 7 (6.7) | 19 (7.0) | 22 (12.7) |  |
| Living with Moderate Frailty, n (%) | 17 (3.1) | 0 (0.0) | 8 (3.0) | 9 (5.2) |  |
| Living with Severe Frailty, n (%) | 4 (0.7) | 1 (1.0) | 2 (0.7) | 1 (0.6) |  |
| Living with Very Severe Frailty, n (%) | 1 (0.2) | 1 (1.0) | 0 (0.0) | 0 (0.0) |  |
| Terminally ill, n (%) | 0 (0.0) | 0 (0.0) | 0 (0.0) | 0 (0.0) |  |
| CHADS_2_ score, points | 3.1 ± 1.3 | 2.4 ± 1.2 | 3.1 ± 1.3 | 3.5 ± 1.2 | **<0.001** |
| CHF, n (%) | 286 (52.2) | 39 (37.5) | 137 (50.6) | 110 (63.6) | **<0.001** |
| Hypertension, n (%) | 442 (80.7) | 84 (80.8) | 214 (79.0) | 144 (83.2) | 0.54 |
| Age, n (%) | 348 (63.5) | 0 (0.0) | 175 (64.6) | 173 (100.0) | **<0.001** |
| Diabetes, n (%) | 204 (37.2) | 39 (37.5) | 96 (35.4) | 69 (39.9) | 0.64 |
| Stroke/TIA, n (%) | 202 (36.8) | 43 (41.3) | 108 (39.9) | 51 (29.5) | 0.050 |
| CHA_2_DS_2_-VASc score, points | 4.7 ± 1.5 | 3.6 ± 1.5 | 4.7 ± 1.5 | 5.2 ± 1.4 | **<0.001** |
| CHF/LV dysfunction, n (%) | 288 (52.6) | 39 (37.5) | 138 (50.9) | 111 (64.2) | **<0.001** |
| Hypertension, n (%) | 442 (80.7) | 84 (80.8) | 214 (79.0) | 144 (83.2) | 0.54 |
| Age over 75, n (%) | 348 (63.5) | 0 (0.0) | 175 (64.6) | 173 (100.0) | **<0.001** |
| Diabetes, n (%) | 204 (37.2) | 39 (37.5) | 96 (35.4) | 69 (39.9) | 0.64 |
| Stroke/TIA, n (%) | 218 (39.8) | 49 (47.1) | 112 (41.3) | 57 (32.9) | 0.050 |
| Vascular disease, n (%) | 169 (30.8) | 22 (21.2) | 86 (31.7) | 61 (35.3) | **0.044** |
| Age between 65 and 74, n (%) | 162 (29.6) | 66 (63.5) | 96 (35.4) | 0 (0.0) | **<0.001** |
| Female gender, n (%) | 163 (29.7) | 24 (23.1) | 73 (26.9) | 66 (38.2) | **0.01** |
| HAS-BLED score, points | 3.2 ± 1.0 | 3.0 ± 1.2 | 3.3 ± 1.0 | 3.2 ± 1.0 | **0.03** |
| Uncontrolled hypertension, n (%) | 60 (10.9) | 12 (11.5) | 34 (12.5) | 14 (8.1) | 0.33 |
| Abnormal renal function, n (%) | 88 (16.1) | 23 (22.1) | 43 (15.9) | 22 (12.7) | 0.12 |
| Abnormal liver function, n (%) | 20 (3.6) | 8 (7.7) | 9 (3.3) | 3 (1.7) | **0.04** |
| Stroke, n (%) | 229 (41.8) | 55 (52.9) | 120 (44.3) | 54 (31.2) | **0.001** |
| Bleeding, n (%) | 348 (63.5) | 58 (55.8) | 175 (64.6) | 115 (66.5) | 0.18 |
| Labile INR, n (%) | 110 (20.1) | 29 (27.9) | 44 (16.2) | 37 (21.4) | 0.04 |
| Elderly, n (%) | 502 (91.6) | 58 (55.8) | 271 (100.0) | 173 (100.0) | **<0.001** |
| Drug abuse, n (%) | 260 (47.4) | 36 (34.6) | 130 (48.0) | 94 (54.3) | **0.006** |
| Alcohol use, n (%) | 147 (26.8) | 34 (32.7) | 74 (27.3) | 39 (22.5) | 0.18 |

Counts with percentages (%) or means ± standard deviations are shown.

Abbreviations: CHF = chronic heart failure; LV = Left ventricular; TIA = Transit ischemic attack.

**Supplemental Table 4 History of bleeding according to age**

|  | **Overall** | **Age <=70** | **70< Age <=80** | **80< Age** | **p-value** |
| --- | --- | --- | --- | --- | --- |
|  | N = 548 | N = 104 | N = 271 | N = 173 |  |
| Intracranial: Epidural, n (%) | 17 (3.1) | 5 (4.8) | 11 (4.1) | 1 (0.6) | 0.06 |
| Intracranial: Subdural, n (%) | 50 (9.1) | 14 (13.5) | 27 (10.0) | 9 (5.2) | 0.06 |
| Gastrointestinal, n (%) | 174 (31.8) | 22 (21.2) | 82 (30.3) | 70 (40.5) | **0.003** |
| Retroperitoneal, n (%) | 6 (1.1) | 0 (0.0) | 3 (1.1) | 3 (1.7) | 0.41 |
| Intraocular, n (%) | 5 (0.9) | 0 (0.0) | 3 (1.1) | 2 (1.2) | 0.55 |
| Respiratory, n (%) | 14 (2.6) | 3 (2.9) | 7 (2.6) | 4 (2.3) | 0.96 |
| Urogenital, n (%) | 26 (4.7) | 4 (3.8) | 13 (4.8) | 9 (5.2) | 0.88 |
| Epistaxis, n (%) | 19 (3.5) | 4 (3.8) | 10 (3.7) | 5 (2.9) | 0.88 |
| Others, n (%) | 40 (7.3) | 8 (7.7) | 22 (8.1) | 10 (5.8) | 0.64 |

Counts with percentages (%) are shown.

**Supplemental Table 5 Comparison between preoperative and intraoperative transesophageal echocardiography assessments according to age**

|  | **Pre-TEE** | **Intraoperative-TEE** | **p-value** |
| --- | --- | --- | --- |
| **Age <=70** |  |  |  |
| LAA ostium diameter 0°, mm | 21.86 ± 3.69 | 20.89 ± 4.07 | **0.02** |
| LAA ostium diameter 45°, mm | 19.81 ± 3.62 | 19.53 ± 3.98 | 0.91 |
| LAA ostium diameter 90°, mm | 20.51 ± 3.86 | 20.10 ± 3.87 | 0.56 |
| LAA ostium diameter 135°, mm | 22.83 ± 4.04 | 21.97 ± 4.11 | 0.11 |
| LAA depth 0°, mm | 28.14 ± 7.96 | 26.47 ± 6.64 | **0.02** |
| LAA depth 45°, mm | 27.97 ± 6.85 | 25.90 ± 7.14 | **0.02** |
| LAA depth 90°, mm | 28.52 ± 7.37 | 26.07 ± 6.97 | **<0.001** |
| LAA depth 135°, mm | 28.52 ± 7.66 | 25.37 ± 6.04 | **<0.001** |
| Spontaneous echo contrast grade 3 or 4, n (%) | 8 (9.8) | 6 (5.8) | 0.25 |
| LAA Thrombus, n (%) | 1 (1.2) | 0 (0.0) | NA |
| Pericardial effusion, n (%) | 4 (4.8) | 7 (6.7) | 0.37 |
| **70< Age <=80** |  |  |  |
| LAA ostium diameter 0°, mm | 21.74 ± 4.02 | 21.48 ± 3.95 | 0.50 |
| LAA ostium diameter 45°, mm | 19.86 ± 3.37 | 19.94 ± 3.71 | 0.60 |
| LAA ostium diameter 90°, mm | 20.57 ± 3.80 | 20.44 ± 4.14 | 0.94 |
| LAA ostium diameter 135°, mm | 22.37 ± 4.07 | 22.32 ± 4.18 | 0.25 |
| LAA depth 0°, mm | 28.53 ± 7.80 | 26.48 ± 6.97 | **<0.001** |
| LAA depth 45°, mm | 27.98 ± 6.94 | 26.80 ± 7.28 | 0.11 |
| LAA depth 90°, mm | 28.60 ± 7.53 | 26.81 ± 7.42 | **0.01** |
| LAA depth 135°, mm | 27.28 ± 6.23 | 25.27 ± 6.22 | **<0.001** |
| Spontaneous echo contrast grade 3 or 4, n (%) | 25 (11.6) | 24 (9.0) | 0.61 |
| LAA Thrombus, n (%) | 1 (0.5) | 1 (0.4) | 1.00 |
| Pericardial effusion, n (%) | 6 (2.8) | 12 (4.4) | **0.041** |
| **80< Age** |  |  |  |
| LAA ostium diameter 0°, mm | 21.57 ± 4.12 | 20.84 ± 4.17 | 0.21 |
| LAA ostium diameter 45°, mm | 20.26 ± 3.77 | 19.76 ± 4.54 | 0.68 |
| LAA ostium diameter 90°, mm | 20.78 ± 4.34 | 19.99 ± 4.20 | 0.12 |
| LAA ostium diameter 135°, mm | 22.42 ± 4.74 | 21.73 ± 4.36 | 0.14 |
| LAA depth 0°, mm | 29.24 ± 7.11 | 25.98 ± 7.19 | **<0.001** |
| LAA depth 45°, mm | 28.62 ± 7.24 | 26.42 ± 6.87 | **<0.001** |
| LAA depth 90°, mm | 28.68 ± 6.62 | 25.95 ± 6.98 | **<0.001** |
| LAA depth 135°, mm | 27.33 ± 7.27 | 25.44 ± 6.69 | **<0.001** |
| Spontaneous echo contrast grade 3 or 4, n (%) | 20 (14.8) | 16 (9.3) | 0.23 |
| LAA Thrombus, n (%) | 1 (0.7) | 0 (0.0) | NA |
| Pericardial effusion, n (%) | 3 (2.2) | 4 (2.3) | 1.00 |

Counts with percentages (%) or means ± standard deviations are shown.

Abbreviations: LAA = Left atrial appendage; NA = Not applicable.

**Supplemental Table 6 Drug regimen according to age**

|  | **Overall** | **Age <=70** | **70< Age <=80** | **80< Age** | **p-value** |
| --- | --- | --- | --- | --- | --- |
|  | N = 548 | N = 104 | N = 271 | N = 173 |  |
| **At baseline** |  |  |  |  |  |
| SAPT, n (%) | 9 (1.6) | 2 (1.9) | 3 (1.1) | 4 (2.3) | 0.60 |
| DAPT, n (%) | 6 (1.1) | 1 (1.0) | 1 (0.4) | 4 (2.3) | 0.16 |
| Warfarin or DOAC, n (%) | 309 (56.4) | 68 (65.4) | 155 (57.2) | 86 (49.7) | **0.04** |
| Warfarin, n (%) | 176 (32.1) | 36 (34.6) | 92 (33.9) | 48 (27.7) | 0.33 |
| Warfarin + any SAPT, n (%) | 82 (15.0) | 15 (14.4) | 43 (15.9) | 24 (13.9) | 0.84 |
| DOAC, n (%) | 352 (64.2) | 66 (63.5) | 172 (63.5) | 114 (65.9) | 0.86 |
| DOAC + any SAPT, n (%) | 123 (22.4) | 18 (17.3) | 56 (20.7) | 49 (28.3) | 0.06 |
| Apixaban, n (%) | 121 (22.1) | 24 (23.1) | 65 (24.0) | 32 (18.5) | 0.38 |
| Dabigatran, n (%) | 29 (5.3) | 8 (7.7) | 15 (5.5) | 6 (3.5) | 0.31 |
| Edoxaban, n (%) | 139 (25.4) | 18 (17.3) | 59 (21.8) | 62 (35.8) | **<0.001** |
| Rivaroxaban, n (%) | 63 (11.5) | 16 (15.4) | 33 (12.2) | 14 (8.1) | 0.16 |
| Triple therapy, n (%) | 14 (2.6) | 1 (1.0) | 10 (3.7) | 3 (1.7) | 0.23 |
| None, n (%) | 0 (0.0) | 0 (0.0) | 0 (0.0) | 0 (0.0) | NA |
| **At discharge** |  |  |  |  |  |
| SAPT, n (%) | 5 (0.9) | 0 (0.0) | 1 (0.4) | 4 (2.3) | 0.06 |
| DAPT, n (%) | 12 (2.2) | 1 (1.0) | 7 (2.6) | 4 (2.3) | 0.63 |
| Warfarin or DOAC, n (%) | 127 (23.2) | 18 (17.3) | 67 (24.7) | 42 (24.3) | 0.29 |
| Warfarin, n (%) | 183 (33.4) | 41 (39.4) | 92 (33.9) | 50 (28.9) | 0.19 |
| Warfarin + any SAPT, n (%) | 155 (28.3) | 36 (34.6) | 77 (28.4) | 42 (24.3) | 0.18 |
| DOAC, n (%) | 349 (63.7) | 64 (61.5) | 171 (63.1) | 114 (65.9) | 0.74 |
| DOAC + any SAPT, n (%) | 234 (42.7) | 50 (48.1) | 108 (39.9) | 76 (43.9) | 0.33 |
| Apixaban, n (%) | 121 (22.1) | 24 (23.1) | 64 (23.6) | 33 (19.1) | 0.51 |
| Dabigatran, n (%) | 28 (5.1) | 7 (6.7) | 14 (5.2) | 7 (4.0) | 0.62 |
| Edoxaban, n (%) | 139 (25.4) | 18 (17.3) | 60 (22.1) | 61 (35.3) | **0.001** |
| Rivaroxaban, n (%) | 61 (11.1) | 15 (14.4) | 33 (12.2) | 13 (7.5) | 0.16 |
| Triple therapy, n (%) | 16 (2.9) | 1 (1.0) | 11 (4.1) | 4 (2.3) | 0.24 |
| None, n (%) | 0 (0.0) | 0 (0.0) | 0 (0.0) | 0 (0.0) | NA |
| **At 45 days** |  |  |  |  |  |
| SAPT, n (%) | 193 (35.4) | 38 (36.9) | 88 (32.7) | 67 (39.0) | 0.39 |
| DAPT, n (%) | 281 (51.7) | 47 (45.6) | 147 (54.6) | 87 (50.6) | 0.28 |
| Warfarin or DOAC, n (%) | 55 (10.1) | 16 (15.5) | 29 (10.8) | 10 (5.8) | **0.02** |
| Warfarin, n (%) | 3 (0.6) | 2 (1.9) | 0 (0.0) | 1 (0.6) | 0.08 |
| Warfarin + any SAPT, n (%) | 6 (1.1) | 1 (1.0) | 5 (1.9) | 0 (0.0) | 0.19 |
| DOAC, n (%) | 29 (5.3) | 7 (6.8) | 16 (5.9) | 6 (3.5) | 0.41 |
| DOAC + any SAPT, n (%) | 17 (3.1) | 6 (5.8) | 8 (3.0) | 3 (1.7) | 0.17 |
| Apixaban, n (%) | 20 (3.7) | 6 (5.8) | 10 (3.7) | 4 (2.3) | 0.33 |
| Dabigatran, n (%) | 6 (1.1) | 2 (1.9) | 3 (1.1) | 1 (0.6) | 0.58 |
| Edoxaban, n (%) | 14 (2.6) | 4 (3.9) | 7 (2.6) | 3 (1.7) | 0.56 |
| Rivaroxaban, n (%) | 6 (1.1) | 1 (1.0) | 4 (1.5) | 1 (0.6) | 0.67 |
| Triple therapy, n (%) | 0 (0.0) | 0 (0.0) | 0 (0.0) | 0 (0.0) | NA |
| None, n (%) | 15 (2.8) | 2 (1.9) | 5 (1.9) | 8 (4.7) | 0.19 |

Counts with percentages (%) or means ± standard deviations are shown.

Abbreviations: DAPT = Dual antiplatelet therapy; DOAC = Direct oral anticoagulant; NA = Not applicable; SAPT = Single antiplatelet therapy.

**Supplemental Table 7** **Transesophageal echocardiography assessments at 45 days follow-up**

|  | **Overall** | **Age <=70** | **70< Age <=80** | **80< Age** | **p-value** |
| --- | --- | --- | --- | --- | --- |
|  | N = 444 | N = 86 | N = 221 | N = 137 |  |
| Device compression 0°, % | 15.3 ± 7.2 | 16.0 ± 7.4 | 15.2 ± 7.2 | 15.1 ± 7.1 | 0.65 |
| Device compression 45°, % | 15.4 ± 6.8 | 16.5 ± 6.3 | 15.5 ± 7.1 | 14.6 ± 6.4 | 0.16 |
| Device compression 90°, % | 15.5 ± 6.6 | 15.6 ± 6.1 | 15.4 ± 7.0 | 15.5 ± 6.2 | 0.98 |
| Device compression 135°, % | 14.9 ± 7.1 | 16.0 ± 6.6 | 14.7 ± 7.0 | 14.7 ± 7.4 | 0.39 |
| Peri-device leak 0°, n (％) |  |  |  |  | 0.53 |
| None | 408 (91.9) | 78 (90.7) | 201 (91.0) | 129 (94.2) |  |
| <3 mm | 26 (5.9) | 6 (7.0) | 16 (7.2) | 4 (2.9) |  |
| 3-5 mm | 3 (0.7) | 0 (0.0) | 1 (0.5) | 2 (1.5) |  |
| >=5 mm | 0 (0.0) | 0 (0.0) | 0 (0.0) | 0 (0.0) |  |
| Peri-device leak 45°, n (％) |  |  |  |  | 0.33 |
| None | 399 (90.9) | 78 (91.8) | 199 (90.9) | 122 (90.4) |  |
| <3 mm | 31 (7.0) | 4 (4.7) | 15 (6.8) | 12 (8.8) |  |
| 3-5 mm | 8 (1.8) | 2 (2.3) | 5 (2.3) | 1 (0.7) |  |
| >=5 mm | 0 (0.0) | 0 (0.0) | 0 (0.0) | 0 (0.0) |  |
| Peri-device leak 90°, n (％) |  |  |  |  | 0.18 |
| None | 384 (86.4) | 70 (81.4) | 189 (85.5) | 125 (91.2) |  |
| <3 mm | 41 (9.2) | 9 (10.5) | 27 (12.2) | 5 (3.6) |  |
| 3-5 mm | 11 (2.5) | 5 (5.8) | 2 (0.9) | 4 (2.9) |  |
| >=5 mm | 1 (0.2) | 0 (0.0) | 1 (0.5) | 0 (0.0) |  |
| Peri-device leak 135°, n (％) |  |  |  |  | 0.15 |
| None | 348 (78.4) | 63 (73.3) | 172 (77.8) | 113 (82.5) |  |
| <3 mm | 72 (16.2) | 16 (18.6) | 37 (16.7) | 19 (13.9) |  |
| 3-5 mm | 18 (4.1) | 5 (5.8) | 10 (4.5) | 3 (2.2) |  |
| >=5 mm | 1 (0.2) | 1 (1.2) | 0 (0.0) | 0 (0.0) |  |
| Device protrusion 0°, mm | 4.1 ± 3.8 | 3.4 ± 3.6 | 4.3 ± 3.7 | 4.3 ± 4.2 | 0.37 |
| Device protrusion 45°, mm | 3.9 ± 3.8 | 2.8 ± 3.3 | 4.1 ± 3.7 | 4.6 ± 4.0 | **0.04** |
| Device protrusion 90°, mm | 5.5 ± 4.2 | 4.6 ± 4.0 | 5.7 ± 4.0 | 5.9 ± 4.5 | 0.23 |
| Device protrusion 135°, mm | 6.0 ± 4.4 | 5.7 ± 4.7 | 6.2 ± 4.3 | 5.9 ± 4.6 | 0.78 |
| Right-left shunt of ASD flow, n (%) | 18 (4.8) | 4 (5.5) | 9 (4.7) | 5 (4.5) | 0.95 |
| Post spontaneous echo contrast, n (%) |  |  |  |  | 0.98 |
| Grade 1 | 100 (24.8) | 20 (27.0) | 48 (23.2) | 32 (26.0) |  |
| Grade 2 | 62 (15.3) | 9 (12.2) | 33 (15.9) | 20 (16.3) |  |
| Grade 3 | 27 (6.7) | 4 (5.4) | 15 (7.2) | 8 (6.5) |  |
| Grade 4 | 3 (0.7) | 1 (1.4) | 1 (0.5) | 1 (0.8) |  |
| Deep device implantation, n (%) | 14 (3.2) | 2 (2.3) | 7 (3.2) | 5 (3.6) | 0.86 |
| DRT, n (%) | 6 (1.4) | 1 (1.2) | 4 (1.8) | 1 (0.7) | 0.68 |
| Thrombus in device, n (%) |  |  |  |  | 0.55 |
| Full | 119 (26.9) | 26 (30.6) | 59 (26.7) | 34 (25.0) |  |
| Half | 95 (21.5) | 18 (21.2) | 51 (23.1) | 26 (19.1) |  |
| Half-none | 83 (18.8) | 19 (22.4) | 41 (18.6) | 23 (16.9) |  |
| None | 145 (32.8) | 22 (25.9) | 70 (31.7) | 53 (39.0) |  |
| Pericardial effusion, n (%) | 8 (1.8) | 2 (2.4) | 5 (2.3) | 1 (0.7) | 0.52 |

Counts with percentages (%) or means ± standard deviations are shown.

Abbreviations: ASD = Atrial septal defect; DRT = Device-related thrombus.

**Supplemental Table 8 Detailed type of postoperative bleeding according to age**

|  | **Overall** | **Age <=70** | **70< Age <=80** | **80< Age** |
| --- | --- | --- | --- | --- |
|  | N = 40 | N = 6 | N = 16 | N = 18 |
| Gastrointestinal bleeding | 13 (32.5) | 2 (5.0) | 6 (15.0) | 5 (12.5) |
| Pericardial effusion | 9 (22.5) | 0 (0.0) | 3 (7.5) | 6 (15.0) |
| Epistaxis | 4 (10.0) | 1 (2.5) | 2 (5.0) | 1 (2.5) |
| Other procedure-related complications* | 4 (10.0) | 2 (5.0) | 2 (5.0) | 0 (0.0) |
| Unexplained anemia | 3 (7.5) | 0 (0.0) | 1 (2.5) | 2 (5.0) |
| Intramuscular | 2 (5.0) | 0 (0.0) | 1 (2.5) | 1 (2.5) |
| Urogenital | 1 (2.5) | 1 (2.5) | 0 (0.0) | 0 (0.0) |
| Intraocular | 1 (2.5) | 0 (0.0) | 0 (0.0) | 1 (2.5) |
| Intraoral | 1 (2.5) | 0 (0.0) | 1 (2.5) | 0 (0.0) |
| TEE-related complication | 1 (2.5) | 0 (0.0) | 0 (0.0) | 1 (2.5) |
| Cancer | 1 (2.5) | 0 (0.0) | 0 (0.0) | 1 (2.5) |

Counts with percentages (%) or means ± standard deviations are shown.

*: Other procedural-related complications were defined procedural-related complications excluding pericardial effusion and TEE-related complication.

Abbreviations: TEE = Transesophageal echocardiography.

**Supplemental Table 9 Drug regimens at the time of bleeding events according to age**

|  | **Overall** | **Age <=70** | **70< Age <=80** | **80< Age** | **p-value** |
| --- | --- | --- | --- | --- | --- |
|  | N = 40 | N = 6 | N = 16 | N = 18 |  |
| DOAC | 10 (25.0) | 1 (2.5) | 5 (12.5) | 5 (12.5) | 0.79 |
| DOAC+SAPT | 13 (32.5) | 3 (7.5) | 4 (10.0) | 5 (12.5) | 0.50 |
| VKA | 4 (10.0) | 1 (2.5) | 2 (5.0) | 1 (2.5) | 0.67 |
| VKA+SAPT | 8 (20.0) | 1 (2.5) | 3 (7.5) | 4 (10.0) | 0.95 |
| Triple Tx | 2 (5.0) | 0 (0.0) | 2 (5.0) | 0 (0.0) | 0.21 |
| SAPT | 3 (7.5) | 0 (0.0) | 0 (0.0) | 3 (7.5) | 0.28 |
| DAPT | 0 (0.0) | 0 (0.0) | 0 (0.0) | 0 (0.0) | NA |
| None | 0 (0.0) | 0 (0.0) | 0 (0.0) | 0 (0.0) | NA |

Counts with percentages (%) or means ± standard deviations are shown.

Abbreviations: DAPT = Dual antiplatelet therapy; DOAC = Direct oral anticoagulant; SAPT = Single antiplatelet therapy; Tx = therapy; VKA = Vitamin K antagonist.
